# Prognostic role and correlation of CA9, CD31, CD68 and CD20 with the desmoplastic stroma in pancreatic ductal adenocarcinoma

**DOI:** 10.18632/oncotarget.12022

**Published:** 2016-09-14

**Authors:** Angela Diana, Lai Mun Wang, Zenobia D'Costa, Abul Azad, Michael A. Silva, Zahir Soonawalla, Paul Allen, Stanley Liu, W. Gillies McKenna, Ruth J. Muschel, Emmanouil Fokas

**Affiliations:** ^1^ Department of Oncology, CRUK/MRC Oxford Institute for Radiation Oncology, University of Oxford, Oxford, UK; ^2^ Department of Pathology, Oxford University Hospital NHS Foundation Trust, Oxford, UK; ^3^ Department of Surgery, Oxford University Hospital NHS Foundation Trust, Oxford, UK; ^4^ Department of Radiation Oncology, Sunnybrook Research Institute, Sunnybrook Health Sciences Centre, University of Toronto, Toronto, Canada; ^5^ Current Address: Department of Radiotherapy and Oncology, Goethe University of Frankfurt, Frankfurt, Germany

**Keywords:** hypoxia, vessel density, macrophages, prognosis, desmoplastic stroma

## Abstract

We assessed the prognostic value of hypoxia (carbonic anhydrase 9; CA9), vessel density (CD31), with macrophages (CD68) and B cells (CD20) that can interact and lead to immune suppression and disease progression using scanning and histological mapping of whole-mount FFPE pancreatectomy tissue sections from 141 primarily resectable pancreatic ductal adenocarcinoma (PDAC) samples treated with surgery and adjuvant chemotherapy. Their expression was correlated with clinicopathological characteristics, and overall survival (OS), progression-free survival (PFS), local progression-free survival (LPFS) and distant metastases free-survival (DMFS), also in the context of stroma density (haematoxylin-eosin) and activity (alpha-smooth muscle actin). The median OS was 21 months after a mean follow-up of 20 months (range, 2–69 months). The median tumor surface area positive for CA9 and CD31 was 7.8% and 8.1%, respectively. Although total expression of these markers lacked prognostic value in the entire cohort, nevertheless, high tumor compartment CD68 expression correlated with worse PFS (*p* = 0.033) and DMFS (*p* = 0.047). Also, high CD31 expression predicted for worse OS (*p* = 0.004), PFS (*p* = 0.008), LPFS (*p* = 0.014) and DMFS (*p* = 0.004) in patients with moderate density stroma. High stromal and peripheral compartment CD68 expression predicted for significantly worse outcome in patients with loose and moderate stroma density, respectively. Altogether, in contrast to the current notion, hypoxia levels in PDAC appear to be comparable to other malignancies. CD31 and CD68 constitute prognostic markers in patient subgroups that vary according to tumor compartment and stromal density. Our study provides important insight on the pathophysiology of PDAC and should be exploited for future treatments.

## INTRODUCTION

Pancreatic ductal adenocarcinoma (PDAC) has a dismal prognosis with a 5-year survival of 5% [1, 2]. Surgery is currently the only potentially curative treatment for PDAC but the majority of patients are diagnosed at advanced inoperable stage, whereas radiotherapy and chemotherapy are associated with high recurrence rates [1, 2]. A common characteristic of PDAC is the presence of a desmoplastic stroma with infiltration by immunosuppressive cells, such as macrophages that has been associated with disease aggressiveness [3, 4], whereas the role of B cells in this disease remains controversial [5–8].

The aberrant vascular architecture of solid tumors in conjunction with increased consumption of oxygen by cancer cells leads to hypoxia that limits the efficacy of conventional treatments and promotes metastasis [9–12]. Traditionally, PDAC has been considered as a highly hypoxic malignancy based on histological studies and pO2 measurements with intratumoral Eppendorf electrode probes [13–16]. However, these studies presented limitations. Indeed, immunohistochemical staining for either carbonic anhydrase (CA9) and hypoxia-inducible factor (HIF-1) has revealed very high expression of these hypoxia markers [17, 18], these analyses were performed in either tissue microarrays (TMAs) or small sections that fail to consider the potentially large tissue heterogeneity across the resected specimen. Similarly, the Eppendorf probe-based measurement of hypoxia by Koong et al. was only conducted in seven patients and hence no definitive conclusions can be drawn [19].

In addition, the stroma of PDAC prevents chemotherapy delivery in part by compromising vascular patency and functionality [13, 20]. Depletion or „re-education“ of the stroma can, in certain conditions, lead to increased vessel density and increased intratumoral gemcitabine concentration, enhancing therapeutic response [13, 20–22], albeit stromal alteration can have mixed effects on tumor progression [23–26]. Also, the accuracy of vessel density assessment in PDAC has been hampered by the examination of small tumor regions, and mixed findings have been reported regarding the prognostic value of vascularity [27–30]. Importantly, previous clinical analyses failed to examine hypoxia and vessel density in the context of desmoplastic stroma [25, 26, 31–33].

We have recently studied the prognostic impact of several important immune markers and cell populations, such as PD-1, PD-L1, CD8 and FOXP3 [34]. In the present work we decided to focus on cell populations that have not been previously investigated on entire pancreatectomy sections. For that purpose, we examine the prognostic impact of macrophages (CD68), B cells (CD20) as well hypoxia (carbonic anhydrase 9; CA9) and vessel density (CD31) alone, and also the correlation with the desmoplastic stroma density and activity based on haematoxylin-eosin and αSMA, respectively, in a large number of patients (*n* = 141) that received primary surgery followed by adjuvant chemotherapy. An additional reason for studying CD68 and C20 is the recent work by Coussens and colleagues which demonstrated that B cell-macrophage interactions lead, via the Bruton tysoine kinase, to immune suppression and PDAC progression [5]. Notably, we performed our study by examining large sections obtained from the entire pancreatectomy sample that takes into consideration the tissue heterogeneity and facilitates a more accurate evaluation of the hypoxic and vascular area distribution and proportion.

## RESULTS

### CA9, CD31, CD68 and CD20 staining characteristics

The median percentage of tumor surface area positive for CA9 and CD31 immunohistochemical staining were 7.8% and 8.1%, respectively. Of note, only 15 (10.6%) and 5 (3.5%) patients had a percentage of positive CA9 tumor area expression higher than 20% and 30%, respectively. The results of CD68 and CD20 immunohistochemistry including the three individual tumor compartment scores (intraepithelial, stroma and periphery) and the total score from all compartments are presented in Table 1.

**Table 1:** Results of CD68 and CD20 immunohistochemistry scoring

Immune marker	CD68 *n* (%)	CD20 *n* (%)
Dichotomized total score*	< 8 vs ≥ 8	< 5 vs ≥ 5
Low expression	65 (46.1)	50 (35.5)
High expression	76 (53.9)	91 (64.5)
Dichotomized tumor (intraepithelial) compartment score	< 2 vs ≥ 2	< 2 vs ≥ 2
Low expression	33 (23.4)	118 (83.6)
High expression	108 (76.6)	23 (16.4)
Dichotomized stroma compartment score	< 3 vs ≥ 3	< 2 vs ≥ 2
Low expression	30 (21.3)	46 (32.6)
High expression	111 (78.7)	95 (67.4)
Dichotomized peripheral compartment score	< 3 vs ≥ 3	< 2 vs ≥ 2
Low expression	52 (36.9)	12 (8.5)
High expression	89 (63.1)	129 (91.5)

* Dichotomized labelling (low vs high expression) based on the median value of immune marker expression. Total score accounted for all three compartment scores (tumor, stroma, periphery).

With regard to the correlation of CA9, CD31, total CD68 and total CD20 expression with the clinicopathological parameters (Table 2), patients younger than 65 years of age (median cut-off) had significantly lower percentages of tumor hypoxia and *vice versa* (low CA9 vs high: *p* < 0.001). Unexpectedly, significantly more patients with vascular invasion (VI) had lower CA9 expression (*p* = 0.034). Also, PNI was more common in patients with high CD68 expression (*p* = 0.016). We failed to detect any further significant correlation between either CA9, CD31, CD68, CD20 and the clinicopathological characteristics (Table 2).

**Table 2:** Clinicopathological characteristics and correlation with CA9, CD31, CD68 and CD20 expression

	Low CA9*n* (%)	High CA9*n* (%)	*p*-value	Low CD31*n* (%)	High CD31*n* (%)	*p*-value	Low CD68*n* (%)	High CD68*n* (%)	*p*-value	Low CD20*n* (%)	High CD20*n* (%)	*p*-value
**Age**												
< median (65 years)	42 (60%)	20 (28.6%)	**< 0.001**	30 (42.9%)	32 (45.7%)	0.734	28 (43.1%)	34 (44.7%)	0.843	22 (44.0%)	46 (50.5%)	0.285
≥ median	28 (40%)	50 (71.4%)		40 (57.1%)	38 (54.3%)		37 (56.9%)	42 (55.3%)		28 (56.0%)	45 (49.5%)	
**Gender**												
Female	34 (48.6%)	34 (48.6%)	1.000	35 (50%)	33 (47.1%)	0.735	29 (44.6%)	39 (51.3%)	0.427	22 (44.0%)	33 (47.1%)	0.457
Male	36 (51.4%)	36 (51.4%)		35 (50%)	37 (52.9%)		36 (55.4%)	37 (48.4%)		28(50%)	37 (52.9%)	
**Tumor site**												
Head	55 (78.6%)	62 (88.6%)	0.111	58 (82.9%)	59 (84.3%)	0.820	56 (86.2%)	62 (81.6%)	0.464	43 (86.0%)	75 (82.4%)	0.843
Other	15 (21.4%)	8 (11.4%)		12 (17.1%)	11 (15.7%)		9 (13.8%)	14 (18.4%)		7 (14.0%)	16 (17.6%)	
**pT-staging**												
pT1-2	42 (60%)	42 (60%)	1.000	44 (62.9%)	40 (57.1%)	0.490	43 (66.2%)	42 (55.3%)	0.188	30 (60.0%)	55 (60.4%)	0.959
pT3-4	28 (40%)	28 (40%)		26 (37.1%)	30 (42.9%)		22 (33.8%)	34 (44.7%)		20 (40.0%)	36 (39.6%)	
**pN-staging**												
pN0	17 (24.3%)	16 (22.9%)	0.842	20 (28.6%)	13 (18.6%)	0.163	17 (26.2%)	17 (22.4%)	0.600	14 (28.0%)	20 (22.0%)	0.424
pN+	53 (75.7%)	54 (77.1%)		50 (71.4%)	57 (81.4%)		48 (73.8%)	59 (77.6%)		36 (72.0%)	71 (78.0%)	
**Grading**												
G1	5 (7.1%)	3 (4.3%)	0.610	4 (5.7%)	4 (5.7%)	0.856	5 (7.1%)	3 (4.3%)	0.610	4 (6.2%)	4 (5.3%)	0.870
G2	42 (60%)	47 (67.1%)		46 (65.7%)	43 (61.4%)		42 (60%)	47 (67.1%)		40 (61.5%)	50 (65.8%)	
G3	23 (35.9%)	20 (28.6%)		20 (28.6%)	23 (32.9%)		23 (35.9%)	20 (28.6%)		21 (32.3%)	22 (28.9%)	
**Resection margins**												
R0	24 (34.3%)	28 (40%)	0.484	24 (34.3%)	28 (40%)	0.484	26 (40.0%)	27 (35.5%)	0.585	16 (32.0%)	57 (40.7%)	0.719
R1	46 (65.7%)	42 (60%)		46 (65.7%)	42 (60%)		39 (60.0%)	49 (64.5%)		34 (68.0%)	54 (59.3%)	
**Type of surgery**												
Whipples	46 (65.7%)	44 (62.9%)	0.833	46 (65.7%)	44 (62.9%)	0.494	44 (67.7%)	46 (60.5%)	0.463	37 (74.0%)	53 (58.2%)	0.119
Pylorus preserving	17 (24.3%)	20 (28.6%)		16 (22.9%)	21 (30.0%)		17 (26.2%)	21 (27.6%)		11 (22.0%)	27 (29.7%)	
Total pancreatectomy	7 (10.0%)	6 (8.6%)		8 (11.4%)	5 (7.1%)		4 (6.2%)	9 (11.8%)		2 (4.0%)	11 (12.1%)	
**PNI**												
no	52 (74.3%)	58 (82.9%)	0.217	59 (84.3%)	51 (72.9%)	0.099	57 (87.7%)	54 (71.1%)	**0.016**	39 (78.0%)	72 (79.1%)	0.876
yes	18 (25.7%)	12 (17.1%)		11 (15.7%)	19 (27.1%)		8 (22.0%)	22 (28.9%)		11 (22.0%)	19 (20.9%)	
**VI**												
no	19 (27.1%)	31 (44.3%)	**0.034**	22 (36.8%)	28 (40%)	0.290	24 (36.9%)	27 (35.5%)	0.863	18 (36.0%)	33 (36.3%)	0.975
yes	51 (72.9%)	30 (55.7%)		48 (68.6%)	42 (60)		41 (63.1%)	49 (64.5%)		32 (64.0%)	58 (63.7%)	
**LI**												
no	23 (32.9%)	28 (40%)	0.380	30 (42.9%)	21 (30%)	0.114	25 (38.5%)	27 (35.5%)	0.719	16 (32.0%)	36 (39.6%)	0.373
yes	47 (67.1%)	42 (60%)		40 (57.1%)	49 (70%)		40 (61.5%)	49 (64.5%)		34 (68.0%)	55 (60.4%)	
**Chemotherapy**												
No	7 (10%)	12 (17.1%)	0.283	6 (8.6%)	13 (18.6%)	0.541	7 (10.8%)	12 (15.8%)	0.523	6 (12.0%)	13 (14.3%)	0.676
1–2 cycles	18 (25.7%)	12 (17.1%)		15 (21.4%)	15 (21.4%)		16 (24.6%)	14 (18.4%)		9 (18.0%)	21 (23.1%)	
≥ 3 cycles	45 (64.3%)	46 (65.7%)		49 (70.0%)	42 (60.0%)		42 (64.6%)	50 (65.8%)		35 (70.0%)	57 (62.6%)	

Abbreviations: PNI, perineural/neural invasion; VI, vascular invasion; LI, lymphatic invation.

**Figure 1:**
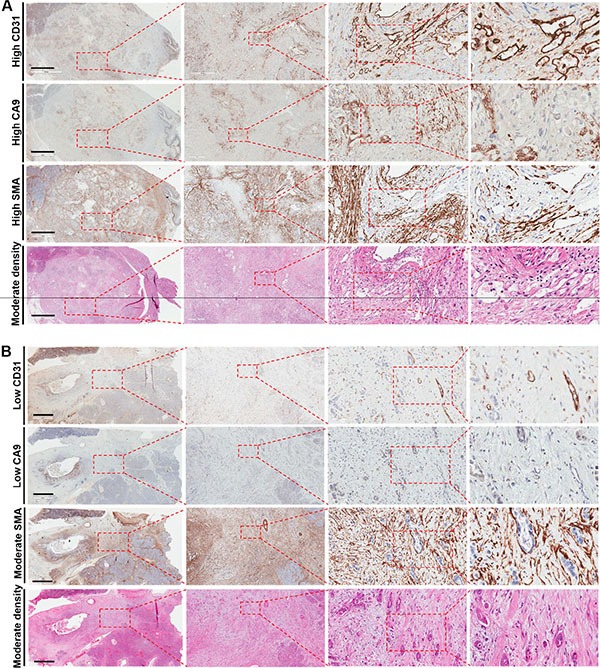
Examples of (A) high CD31 and CA9 expression and (B) low CD31 and CA9 expression in patients with pancreatic cancer adenocarcinoma The corresponding haematoxyllin-eosin (stroma density) and αSMA (stroma activation) images are shown as well. The left panels illustrate large pancreatectomy sections (Bar: 6 mm). The magnifications of the second, third and fourth inserts from the left are × 50, × 200 and × 400, respectively.

**Figure 2:**
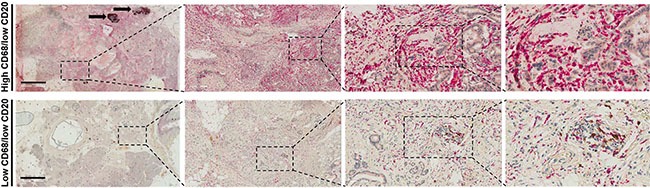
Examples of (A) high CD68 expression and (B) low CD68 expression in patients with pancreatic cancer adenocarcinoma The corresponding CD20 images are shown as well. The left panels illustrate large pancreatectomy sections (Bar: 6 mm). Black arrows indicate two lymph node metastases. The magnifications of the second, third and fourth inserts from the left are × 50, × 200 and × 400, respectively.

Image examples of whole-mount pancreatectomy sections with low and high CA9 and CD31 together with the corresponding H&E (stromal density), αSMA (stromal activation) images are shown in Figure 1. Scanning and histological mapping revealed interpatient and intrapatient heterogeneity with regard to the extent and the localization of regions with positive CA9 and CD31 staining. This finding highlights the importance of using large pancreatectomy sections rather than TMAs or small sections as the latter can lead to either under- or overestimation of marker expression. Examples of images with high and low CD68 expression with the corresponding CD20 (double staining) are shown in Figure 2. The clinicopathological characteristics for the entire cohort are shown in Supplementary Table 1.

### Hypoxia, vessel density, immune markers and prognosis

The median OS for the entire cohort was 21 months and the 3-year OS was 36% after a mean follow-up of 20 months (range, 2–69 months). From the entire cohort, 55 (39%) patients presented with distant recurrence, 15 (10.6%) developed local recurrence, 14 (9.9%) had both local and distant recurrence, whereas 57 (40.4%) lacked any recurrence by the time of analysis. In the univariate analysis, CA9 lacked any statistical prognostic significance for either OS (*p* = 0.229), PFS (*p* = 0.388), LPFS (*p* = 0.0.375) or DMFS (*p* = 0.405) (Table 3). Similarly, CD31, total CD68 and total CD20 expression failed to demonstrate any correlation with the clinical outcome. Advanced T-stage (T3-4 vs T1-2), the presence of lymph node metastases (pN+ vs pN0), resection margin status, perineural/neural invasion (PNI), venous invasion (VI) and chemotherapy adversely affected all four clinical endpoints. High tumor grading negatively impacted PFS (*p* = 0.009) and DMFS (*p* = 0.004) but not OS (*p* = 0.056) or LPFS (*p* = 0.064). We failed to detect a significant correlation for either sex, age, tumor localization or type of surgery with the clinical outcome (Table 3).

**Table 3:** Univariate and multivariate analysis of prognostic factors in the entire patient cohort (*n* = 141)

	Univariate		Multivariate		
	*p*-value	HR	95% CI		*p*-value
			Lower	Upper	
**OS**					
CA9 (Low vs High)	0.229	1.207	0.720	2.022	0.475
CD31 (Low vs High)	0.285	0.956	0.583	1.569	0.860
CD68 (Low vs High)	0.623	1.032	0.860	1.237	0.738
CD20 (Low vs High)	0.864	1.048	0.637	1.724	0.854
Age (< median(65) vs ≥ median)	0488	1.180	0.702	1.985	0.532
Sex (male vs female)	0.291	1.176	0.722	1.917	0.514
Tumour localisation (head vs other)	0.204	0.633	0.279	1.436	0.274
pT-stage (pT1-2 vs pT3-4)	**0.001**	0.495	0.295	0.829	**0.008**
pN-stage (pN0 vs pN+)	0.001	1.874	0.927	3.786	0.080
Grading (G1 vs G2 vs G3)	0.056	1.252	0.815	1.926	0.305
Resection margins (R0 vs R1)	0.001	1.374	0.769	2.453	0.283
Type of surgery (W vs PP vs TP)	0762	1.170	0.745	1.839	0.496
PNI (no vs yes)	**0.001**	1.695	0.977	2.941	0.060
VI (no vs yes)	**0.010**	1.633	0.883	3.023	0.118
LI (no vs yes)	0.11	0.923	0.516	1.650	0.787
Chemotherapy (no vs 1-2 cycles vs ≥ 3 cycles)	**< 0.001**	0.564	0.406	0.782	**0.001**
**PFS**					
CA9 (Low vs High)	0.388	1.100	0.680	1.780	0.697
CD31 (Low vs High)	0.349	0.894	0.569	1.406	0.628
CD68 (Low vs High)	0.745	1.010	0.849	1.202	0.908
CD20 (Low vs High)	0.623	0.912	0.586	1.419	0.683
Age (< median(65) vs ≥ median)	0.532	1.168	0.713	1.914	0.538
Sex (male vs female)	0.790	1.041	0.661	1.638	0.864
Tumour localisation (head vs other)	0.204	0.700	0.343	1.429	0.327
pT-stage (pT1-2 vs pT3-4)	**0.002**	0.557	0.350	0.887	**0.014**
pN-stage (pN0 vs pN+)	**0.001**	2.537	1.309	4.916	**0.006**
Grading (G1 vs G2 vs G3)	**0.009**	1.476	0.991	2.197	0.055
Resection margins (R0 vs R1)	**0.001**	1.354	0.798	2.300	0.261
Type of surgery (W vs PP vs TP)	0.704	1.022	0.691	1.510	0.915
PNI (no vs yes)	**< 0.001**	1.878	1.111	3.177	0.019
VI (no vs yes)	0.007	1.473	0.854	2.542	0.164
LI (no vs yes)	0.077	0.961	0.576	1.605	0.880
Chemotherapy (no vs 1-2 cycles vs ≥ 3 cycles)	**< 0.001**	0.692	0.509	0.941	**0.019**
**LPFS**					
CA9 (Low vs High)	0.375	1.130	0.682	1.874	0.634
CD31 (Low vs High)	0.252	1.015	0.630	1.636	0.950
CD68 (Low vs High)	0.446	1.046	0.874	1.252	0.625
CD20 (Low vs High)	0.850	1.011	0.631	1.622	0.962
Age (< median(65) vs ≥ median)	0.222	1.414	0.845	2.367	0.187
Sex (male vs female)	0.435	1.276	0.805	2.021	0.299
Tumour localisation (head vs other)	0.186	0.689	0.318	1.494	0.345
pT-stage (pT1-2 vs pT3-4)	**0.007**	0.612	0.375	1.000	**0.050**
pN-stage (pN0 vs pN+)	**0.001**	1.930	0.980	3.799	0.057
Grading (G1 vs G2 vs G3)	0.064	1.241	0.824	1.868	0.301
Resection margins (R0 vs R1)	**0.001**	1.496	0.858	2.609	0.156
Type of surgery (W vs PP vs TP)	0.814	1.056	0.687	1.624	0.804
PNI (no vs yes)	**< 0.001**	1.960	1.139	3.374	**0.015**
VI (no vs yes)	**0.008**	1.543	0.851	2.796	0.153
LI (no vs yes)	**0.011**	0.937	0.541	1.622	0.816
Chemotherapy (no vs 1-2 cycles vs ≥ 3 cycles)	**< 0.001**	0.606	0.442	0.830	**0.002**
**DMFS**					
CA9 (Low vs High)	0.405	1.189	0.728	1.944	0.489
CD31 (Low vs High)	0.342	0.880	0.550	1.408	0.594
CD68 (Low vs High)	0.949	0.965	0.810	1.150	0.688
CD20 (Low vs High)	0.526	0.848	0.536	1.341	0.481
Age (< median(65) vs ≥ median)	0.818	1.042	0.628	1.729	0.874
Sex (male vs female)	0.452	1.119	0.701	1.786	0.637
Tumour localisation (head vs other)	0.291	0.674	0.318	1.428	0.303
pT-stage (pT1-2 vs pT3-4)	**0.001**	0.409	0.250	0.671	**0.001**
pN-stage (pN0 vs pN+)	**0.001**	2.347	1.197	4.600	**0.013**
Grading (G1 vs G2 vs G3)	**0.004**	1.485	0.982	2.243	0.061
Resection margins (R0 vs R1)	**0.001**	1.351	0.779	2.342	0.284
Type of surgery (W vs PP vs TP)	0.428	1.010	0.666	1.530	0.963
PNI (no vs yes)	**0.001**	1.590	0.926	2.730	0.093
VI (no vs yes)	**0.005**	1.640	0.933	2.882	0.086
LI (no vs yes)	0.078	0.963	0.568	1.634	0.890
Chemotherapy (no vs 1-2 cycles vs ≥ 3 cycles)	**< 0.001**	0.706	0.510	0.976	**0.035**

Abbreviations: HR. hazard ratio; CI, confidence interval; W, Whipples; PP, partial pancreatectomy; TP, total pancreatectomy; VI, vascular invasion; LI, lymphatic invasion; PNI, perineural/neural invasion; OS, overall survival; PFS, progression-free survival; LFFS, local failure-free survival; DMFS, distant metastases-free survival;

Significant values have been marked with bold.

Subsequently, we performed a multivariate analysis by including CA9, CD31, CD68, CD20 and the clinicopathological factors (Table 3). In the Cox model adjuvant chemotherapy and advanced T-stage (pT3-4 vs pT1-2) retained their significance for all four clinical endpoints. The presence of lymph node metastases (pN+ vs pN0) correlated with worse PFS (*p* = 0.006) and DMFS (*p* = 0.013), whereas PNI was associated with worse PFS (*p* = 0.019) and LPFS (*p* = 0.015).

**Figure 3:**
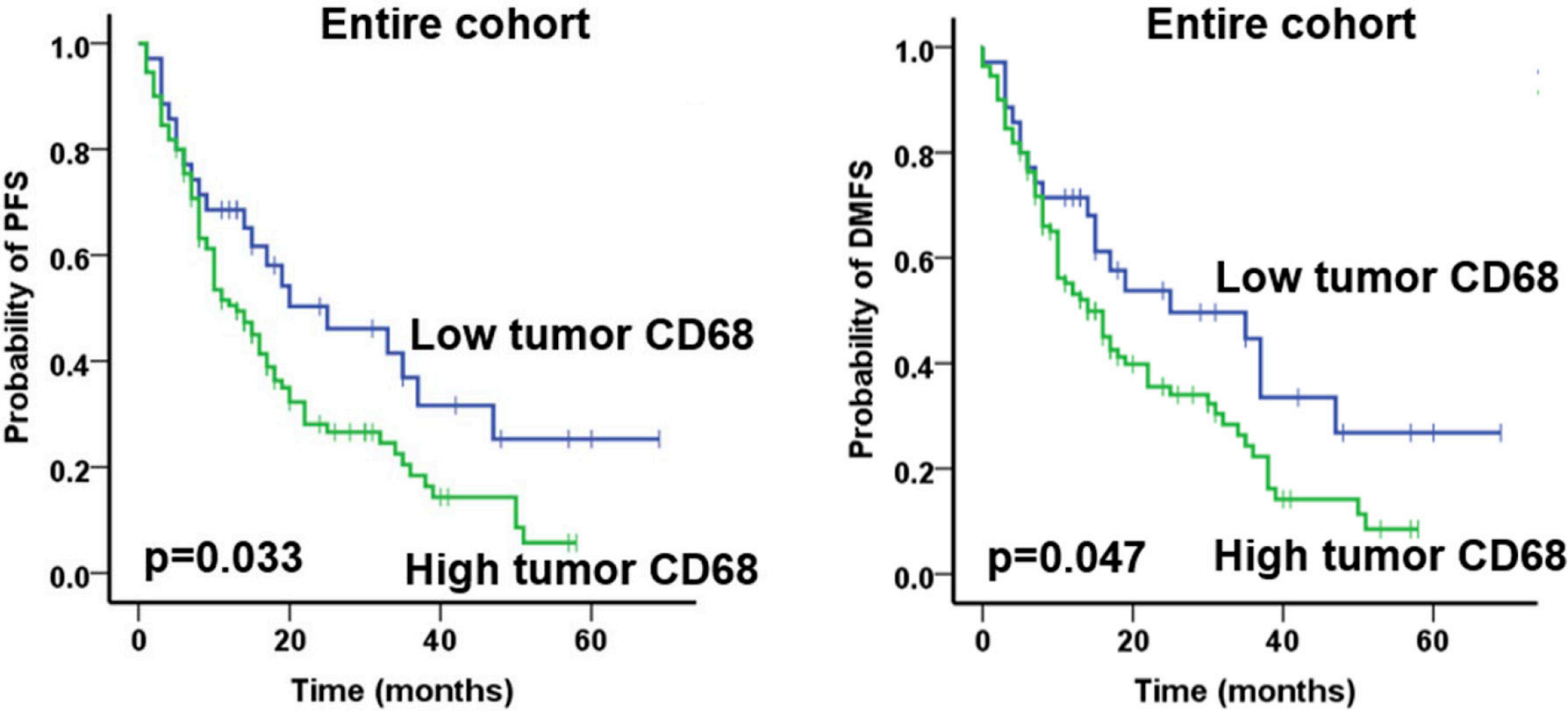
Prognostic impact of tumor compartment CD68 expression on progression-free survival (PFS) and and distant metastases free survival (DMFS) in the entire cohort, as indicated Only significant data are shown here. Analysis was based on the dichotomized tumor compartment CD68 score in resected patient samples (cut-off according to median value of tumor compartment score).

We conducted a separate analysis whereby we examined the prognostic role of the markers according to the different tumour compartments. Only high tumor (intraepithelial) compartment expression of CD68 predicted for worse PFS (*p* = 0.033) and DMFS (*p* = 0.047) (Figure 3), whereas no other significant findings were found for CD20.

### The correlation of hypoxia, vessel density and immune markers with stromal morphology

We have recently demonstrated that the desmoplastic stroma of human PDAC is not homogeneous but rather presents three different patterns of stroma density and activation [31]. Patients with high stromal density tumors had a significantly superior outcome compared to patients with moderate or loose density [31]. Because the lack of prognostic value for CA9, CD31, total CD68 and total CD20 expression was an unexpected finding, we first examined the correlation of these markers with stromal density (H&E) and stromal activation (αSMA; Supplementary Tables 2–3). Interestingly, tumors with high stromal density had lower levels of CA9 expression and, *vice versa*, tumors with loose stroma had higher CA9 expression (*p* = 0.037). CD31 expression did not show variation with stromal density. Similarly, there was no correlation of either CA9 or CD31 with αSMA (Supplementary Table 2), or for CD68 and CD20 with the stromal morphology (Supplementary Table 3).

**Figure 4:**
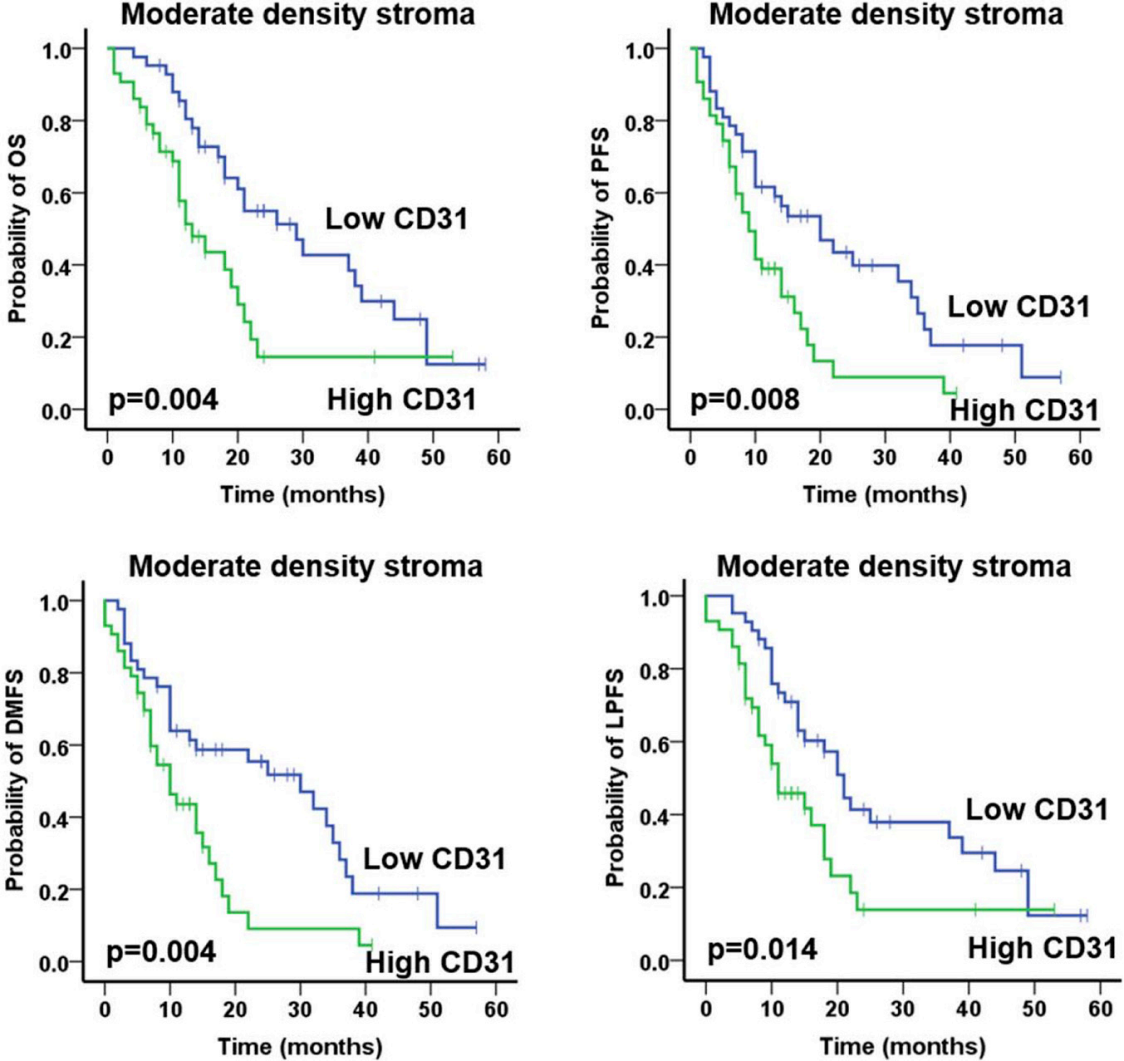
Prognostic impact of total CD31 expression on overall survival (OS), progression-free survival (PFS), local progression-free survival (LPFS) and distant metastases free survival (DMFS) in patients with tumors of moderate stroma density, as indicated Analysis was based on the dichotomized percentage of CD31 surface area expression in resected patient samples (cut-off according to median value of surface area expression percentage).

Subsequently, we investigated the prognostic impact of the CA9 and CD31 according to the different degrees of stromal density and activation (Table 4). Intriguingly, in contrast to the entire cohort, patients with high CD31 expression in the moderate stromal density subgroup had a significantly worse OS (low vs high CD31: mean 30.2 vs 18.3 months; *p* = 0.004), PFS (low vs high CD31: mean 23.7 vs 12.3 months; *p* = 0.008), LPFS (low vs high CD31: mean 27.2 vs 16.8 months; *p* = 0.014) and DMFS (low vs high CD31: mean 26.1 vs 12.6 months; *p* = 0.004) (Figure 4; Table 4).

**Table 4:** Prognostic impact of CA9 and CD31 according to stroma density and activation

Marker expression (high vs low)	OS *p*-value	PFS *p*-value	LPFS *p*-value	DMFS *p*-value
CA9				
Dense stroma	0.715	0.517	0.775	0.860
Moderate stroma	0.913	0.617	0.879	0.756
Loose Stroma	0.658	0.985	0.667	0.889
CD31				
Dense stroma	0.303	0.163	0.250	0.263
Moderate stroma	**0.00**4	**0.008**	**0.014**	**0.004**
Loose Stroma	0.415	0.147	0.772	0.162
CA9				
Absent/low SMA	0.420	0.542	0.283	0.791
Moderate/strong SMA	0.702	0.821	0.905	0.681
CD31				
Absent/low SMA	0.702	0.338	0.462	0.241
Moderate/strong SMA	0.258	0.432	0.285	0.520

Abbreviations: OS, overall survival; PFS, progression-free survival; LFFS, local failure-free survival; DMFS, distant metastases-free survival; significant values have been marked with bold.

Similarly, we analysed the prognostic role of CD68 and CD20 according to the three tumor compartments (tumor, stromal and peripheral) separately, and also with regard to stromal morphology (Table 5; Supplementary Table 4; Supplementary Figure 1). High stromal CD68 expression predicted for significantly worse PFS (*p* = 0.017) and DMFS (*p* = 0.023) in patients with tumors of lose density, whereas high peripheral compartment CD68 expression correlated with less favourable PFS (*p* = 0.013), LPFS (*p* = 0.044) and DMFS (*p* = 0.048) only in patients with moderate density tumors. We did not observe any further significant differences in the prognostic role of these immune markers according to the tumor compartment.

**Table 5:** Prognostic impact of CD68 and CD20 according to stroma density

Marker expression (high vs low)	OS *p*-value	PFS *p*-value	LPFS *p*-value	DMFS *p*-value
Total CD68				
Dense stroma	0.725	0.954	0.509	0.804
Moderate stroma	0.307	0.102	0.096	0.940
Loose Stroma	0.399	0.256	0.988	0.283
Total CD20				
Dense stroma	0.736	0.701	0.753	0.804
Moderate stroma	0.373	0.955	0.975	0.940
Loose Stroma	0.366	0.269	0.199	0.333
Stromal compartment CD68				
Dense stroma	0.370	0.884	0.439	0.964
Moderate stroma	0.454	0.185	0.305	0.243
Loose Stroma	0.331	**0.017**	0.182	**0.023**
Stromal compartment CD20				
Dense stroma	0.473	0.196	0.210	0.235
Moderate stroma	0.647	0.827	0.957	0.834
Loose Stroma	0.214	0.514	0.333	0.536
Tumor compartment CD68				
Dense stroma	0.113	0.126	0.133	0.196
Moderate stroma	0.292	0.063	0.105	0.069
Loose Stroma	0.748	0.775	0.496	0.796
Tumor compartment CD20				
Dense stroma	N/A	N/A	N/A	N/A
Moderate stroma	0.678	0.981	0.609	0.823
Loose Stroma	N/A	N/A	N/A	N/A
Peripheral compartment CD68				
Dense stroma	0.432	0.398	0.460	0.559
Moderate stroma	0.065	**0.013**	**0.044**	**0.048**
Loose Stroma	0.153	0.985	0.372	0.989
Peripheral compartment CD20				
Dense stroma	0.483	0.544	0.089	0.097
Moderate stroma	0.785	0.189	0.375	0.272
Loose Stroma	0.393	0.761	0.693	0.601

Abbreviations: OS, overall survival; PFS, progression-free survival; LFFS, local failure-free survival; DMFS, distant metastases-free survival; significant values have been marked with bold.

### Immune markers and lymphoid aggregates

In total, 57 (40.4%) patients from the entire cohort (*n* = 141) presented with intratumoral lymphoid aggregates, based on H&E staining. Hence, we examined the expression (absent vs present) of CD68 and CD20 in the lymphoid aggregates and their clinical impact (Supplementary Table 5). The presence of CD68 and CD20 did not correlate with the clinical outcome. Of note, the majority of B cells were located in lymphoid aggregates in close association with macrophages, whereas B cell infiltration throughout the tumor area was only scarce in our series (Supplementary Figure 2).

## DISCUSSION

Although previous studies have analyzed the prognostic role of hypoxia markers HIF-1α and CA9, and CD31-based vascular density in patients with PDAC, the vast majority had been performed using either TMAs or small sections, whereas the correlation with the desmoplastic stroma remains, to the best of our knowledge, unexplored. Here, we examined hypoxia and vessel density using CA9 and CD31, respectively, in whole-mount pancreatectomy sections. We observed interpatient and intrapatient variability in the distribution of hypoxia throughout the tumor surface area. More importantly, and in contrast to the notion that PDAC is a highly hypoxic malignancy, a large proportion of tumor samples presented with either minimal or even lack of CA9 expression in our cohort. Indeed, from the *n* = 141 samples of the entire cohort, only 15 (10.6%) and 5 (3.5%) patients had a percentage of positive CA9 tumor surface area expression higher than 20% and 30%, respectively. Our data are in line with and further build upon a recent prospective clinical study in *n* = 10 patients that received intravenously the hypoxia marker pimonidazole preoperatively [35]. In that work, seven patients had minimal or very low levels of hypoxia, whereas the most hypoxic tumors showed positive pimonidazole staining in 20-30% of tumor surface area. Also, in accordance to our observation, large variability was noted [35].

Several reasons could be responsible for discrepancy between our results and previous reports indicating that PDAC is severely hypoxic [13–16, 19]. First, the measurement of hypoxia in clinical samples is challenging as different methods have been proposed including staining with nitroimidazoles, HIF-1α and CA9, pO2 assessment with Eppendorf probes or even imaging with F-MISO PET-CT scan [13–16, 19]. Second, the vast majority of studies have used small sections that failed to consider the substantial intratumoral heterogeneity of PDAC [17, 18]. Third, in contrast to our analysis that was conducted using automated computerized slide scanning and analysis of hypoxia expression, previous histological studies have used manual scoring of hypoxia which can be biased and is far from optimal for histological quantification of hypoxia. Our findings could have implications for strategies to alleviate hypoxia. For example, recent preclinical and clinical work using the prodrug TH-302 that releases the DNA alkylator bromo-isophosphoramide mustard in hypoxic areas demonstrated promising results [36, 37]. However, as we show in the present work, several patients had minimal or undetectable hypoxia levels in their tumors and hence caution is needed as this agent is unlikely to be effective in all patients with PDAC.

PDAC has been considered a hypovascular and poorly perfused tumor, albeit large scale imaging studies in the clinical setting are still lacking [13, 20]. We and others have recently described differential survival rates in patients with PDAC that varied significantly according to the degree of desmoplastic stromal density (H&E) and activation (αSMA) [31, 32]. In our series, tumors exhibited heterogeneous distribution of CD31-positive blood vessels throughout the tumor area, whereas vascularity in adjacent normal pancreas appeared less heterogeneous. High vessel density was significantly associated with worse clinical outcome only in patients with tumors of moderate but not strong or loose stroma density, whereas no correlation was found according to stromal activation. This intriguing finding could, in part, explain the discrepancy in reports on the prognostic role of vessel density. Indeed, some groups have described a strong prognostic impact, while other reports have failed to identify a correlation of vessel density with survival [27–30, 38]. Of note, angiogenesis inhibitors failed to demonstrate efficacy in PDAC [4]. Also, preclinical studies in transgenic mouse models of PDAC have shown that depletion or “re-education” of the stroma can reduce solid stress and/or interstitial fluid pressure to decompress blood vessels to enhance chemotherapy delivery and improve survival [20]. Depletion of stroma in sonic hedgehog-deficient mice led to more aggressive tumors that responded to blockade of vascular endothelial growth factor (VEGF) blockade [23]. Thus, our data on the adverse role of vessel density only in patients with moderately-dense stroma should be explored in larger series as the concept of vascular remodeling using either direct VEGF-blocking agents [10] or indirectly with agents such as PI3K/mTOR inhibitors [39] with the aim of normalizing vasculature making chemotherapy and radiotherapy more efficacious might still be applicable in a subgroup of patients.

Macrophages have been classified into two groups depending on their functional status, that is classically activated (M1) and alternatively activated (M2) macrophages. In malignancies, tumor associated macrophages (TAMs) have been traditionally considered to belong to the polarized M2 phenotype and can be identified using varies markers, such as CD163, CD23, IL-10, CXCR2 and others [40] M2-type TAMs decrease response to chemotherapy and radiotherapy, impair T cell infiltration and function, and promote immune evasion and tumor progression [40, 41]. Previous groups have investigated the prognostic impact of macrophages and reported an adverse impact on outcome [42, 43] but their association with the desmoplastic stroma in human samples remains unexplored. We failed to detect a significant clinical role for total CD68 expression. Instead, high tumor compartment CD68 expression predicted for worse outcome in the entire cohort, similar to stromal compartment CD68 infiltration in loose density tumors. Thus, immune markers should be examined in the context of tumor compartment and desmoplastic stroma, as analysis of their total expression in isolation can “mask” their prognostic impact in patient subgroups. These data are also important with the progressively increased testing of agents targeting macrophages, such as colony-stimulating factor 1 (CSF1) inhibitors [44] in the clinical setting.

Moreover, we did not find a prognostic significance for B cells in our series. B cells constitute effector cells and mediate cellular immunity via antigen-presentation, promoting tumor-specific activation of cytotoxic T cells [45] but their prognostic role has been controversial. Castino et al. recently demonstrated that the vast majority of B cells were located in lymphoid aggregates in close association with T cells and TAMs, as in our present series [8]. High B cell expression in lymphoid aggregates correlated with a better outcome. In contrast, expression throughout the rest of the tumor surface was only scarce, and stromal infiltration failed to predict for outcome [8]. Recently, three preclinical studies reported a protumorigenic role for different B cell subpopulations via diverse mechanisms in PDAC [5–7]. Thus, it is likely that B cells can exert both pro- and antitumorigenic roles in PDAC, depending on the pathophysiological context and possibly tumor localization, highlighting their complexity.

Lymphoid aggregates constitute lymphoid-like structures that vary from T and B cells clusters to germinal-like centers, and have been previously described in cancer, infection and autoimmune diseases [46]. Mixed findings have been reported regarding their prognostic value [46]. In total *n* = 57 patients presented with lymphoid aggregates in our cohort. A recent study by Lutz et al. demonstrated lack of lymphoid aggregates in 54 previously untreated patients that received primary surgical resection [47], which is in contrast to our present findings. In that work, Lutz et al. found aggregates in 33 out of the 39 (84.6%) patients 2 weeks after administration of a GVAX vaccine [47]. The presence of either CD68+ or CD20+ cells in lymphoid aggregates did not possess a prognostic role in our series. Further reports on the impact of lymphoid aggregates in PDAC are lacking, possibly due to the fact that the majority of pathological investigations have been performed in TMAs rather than whole-mount sections.

We would like to acknowledge the limitations of our work. First, although patients were treated and followed up prospectively, the retrospective nature cannot exclude potential selection bias. Second, the median follow-up in our study is relatively short. Also, CD68 is a pan-macrophage marker, which does not provide any information with regard to the polarization status of macrophages, such as CD163 that is specific for M2-macrophages. Furthermore, the lack of automated confirmation of CD68 and CD20 constitutes another limitation. Finally, our observations warrant validation in prospective cohorts, preferably in large pancreatectomy sections.

In conclusion, in contrast to the current belief that PDAC is severely hypoxic, examination of entire pancreatectomy sections demonstrated hypoxia levels comparable to those described in others malignancies. Histological mapping of CA9 and CD31 staining showed inter- and intrapatient heterogeneity. Although CD31 and CD68 failed to demonstrate a prognostic role in the entire cohort, analysis revealed an adverse impact in the outcome of patient subgroups that varied with the tumor compartment and stromal density. Our findings provide important insight on the pathophysiology of PDAC and could be potentially exploited in future studies to guide novel therapeutics.

## MATERIALS AND METHODS

### Patients and treatment

During the period between 2009 and 2014 patients with previously-untreated PDAC received surgery followed by adjuvant chemotherapy at the Oxford University Hospital NHS Trust, Oxford, UK. The type of pancreatectomy performed was conducted according to international guidelines. Patients included in the present retrospective study had to meet the following criteria: histologically-confirmed PDAC, complete macroscopic surgical resection (R0 or R1), lack of metastatic spread and/or ascites, absence of previous history of malignancy, archived formalin-fixed paraffin-embedded (FFPE) surgical samples at the Department of Pathology. In total, *n* = 141 patients were included in the present study. The majority of patients were treated with adjuvant chemotherapy consisted of gemcitabine (GEM) as monotherapy. GEM alone was administered intravenously (dose 1,000 mg/m^2^ over 30 minutes), at days 1, 8 and 15 (1 cycle) for up to 6 cycles. Few patients received a combination of gemcitabine with capecitabine (GEM-CAP). In this case, GEM was administered as described above and CAP was administered orally (dose 830 mg/m^2^ twice daily) for 3 weeks followed by 1-week pause. FFPE tissue blocks were obtained from the Department of Pathology archive together with clinical follow-up data and diagnostic images at the Oxford University Hospital NHS Trust. Patients had previously provided an informed consent. The present study was approved by the Oxford Radcliffe Biobank, University of Oxford (Project: OCHRe 14/A176).

### Immunohistochemical staining and scoring

An experienced gastrointestinal pathologist (LMW) reviewed the *n* = 141 pancreatomy samples. For the purpose of the present study, the best representative FFPE tissue block according to the following criteria: most representative of stromal morphology, least amount of necrosis highest cellularity. Sections (3-μm thick) were cut and mounted on coated superfrost slides. Slides were stained with haematoxylin and eosin (H&E) as previously reported [48]. The Leica Bond Max staining platform was used for the immunohistochemical studies at the Department of Pathology, Oxford University Hospital NHS Trust in conjuction with a DAKO Autostainer Link 48 (DAKO, UK) using a horseradish-peroxidase technique. The Leica DS 9800 detection system facilitated antibody detection. Automatic antigen retrieval was conducted by the pretreatment of the paraffin sections (SuperFrost Plus, Thermo Scientific, UK) with either Bond ER 1 (Citrate based buffer at Ph 6) or Bond ER2 (EDTA based buffer at Ph 9; both Leica Microsystems, UK) for 20 min on the Bond Max staining machine. After that, staining with the primary antibodies for CA9 (1:200; Leica, UK), CD31 (1:80 Dako, UK) was performed after incubation for 20 minutes on the Bond staining platform. Double staining for CD68 (Dako, UK) and CD20 (Dako, UK) was performed in a similar manner. Subsequently, slides were stained with dextran polymer-conjugated horseradish-peroxidase and 3,3′-diaminobenzidine (DAB) chromogen intensified with 1 % copper sulphate followed by a light haematoxylin counterstain (Gill 3, Sigma, UK).

To take into account the intratumoral heterogeneity and facilitate precise calculation of the percentage of surface area positive for CA9 and CD31 staining, the large pancreatectomy sections were scanned by Aperio ScanScope XT at × 20 magnification. We used the Positive Pixel Count Algorithm of the ImageScope Viewer (Aperio Technologies, Inc., Vista, CA, USA) to analyse the percentage of the tissue surface area that was positive for CA9 and CD31 in the large pancreatectomy sections. To investigate the prognostic role of hypoxia and vessel density, we used the median score value as a cut-off to classify patients into two groups: low or high CA9 and CD31 expression. Additionally, we examined the prognostic value of CA9 and CD31 in the context of the desmoplastic stroma density and activation. For that purpose, stromal density was assessed based on haematoxyllin-eosin staining (HE) into loose, moderate or strong stroma, whereas αSMA (stromal activation) was scored as high, moderate and low as recently reported [31].

The expression of CD68 and CD20 was assessed semiquantitatively by measuring cell density as previously reported [49, 50]. Immunohistochemical scoring was conducted as follows: (1) absent cells; (2) < 25% cell density; (3) 25–50% cell density; (4) > 50% cell density. Cells were assessed in all three compartments of the tumor: the intra-epithelial compartment (cells within and in direct contact with tumor cell nests); the stroma (cells within the intratumoral stroma) and the tumor periphery (cells localised in tumor periphery). The sum of the separate scores from the three tumor compartments (tumor, stromal and peripheral compartment) determined the total score for CD68 and CD20. The total score ranged from 3 to 12. The median score value was used as a cut-off to separate patients into two groups: low or high CD68 and CD20 expression. In addition, we examined the prognostic impact of CD68 and CD20 for each of the 3 different compartments separately. The median score of each area was measured and the cut-off point was chosen to separate the cohort into two subgroups with either low or high score. Stromal density was defined as loose, moderate or strong, whereas αSMA was classified as high, moderate [31]. Finally, the presence or absence of CD68 and CD20 expression was considered for estimating their prognostic value in lymphoid aggregates.

### Statistics

The differences between categorical variables were anlysed with the Fisher's exact test. Overall survival (OS) was calculated from the date of surgery to the day of death from any cause. Progression-free survival (PFS) was measured from the date of surgery to the day of local or distant recurrence, or death from any cause. Distant metastasis free survival (DMFS) and local progression free survival (LPFS) were assessed from the date of surgery to distant metastasis or death and local progress or death, respectively. Patients that lacked local or distant tumor recurrence were censored at the time of the last follow-up. A *p-value* lower than 0.05 was considered as statistically significant. The Kaplan-Meier method was used to plot the survival curves. Univariate analyses were made with the log-rank (Mantel–Cox) test and multivariate analyses with the Cox proportional hazard model. All statistical analyses were performed with the SPSS 20 software (SPSS Inc., Chicago, IL, USA).

## SUPPLEMENTARY MATERIALS FIGURES AND TABLES


