# Supplementary material for: Prognostic role and correlation of CA9, CD31, CD68 and CD20 with the desmoplastic stroma in pancreatic ductal adenocarcinoma
Source: Oncotarget. 2016 Sep 14;7(45):72819–32. doi: 10.18632/oncotarget.12022 (PMC5341946; doi:10.18632/oncotarget.12022)
Supplement: Supplementary file 1 [file oncotarget-07-72819-s001.pdf]

Prognostic role and correlation of CA9, CD31, CD68 and CD20 with the desmoplastic stroma in pancreatic ductal adenocarcinoma

Supplementary Materials

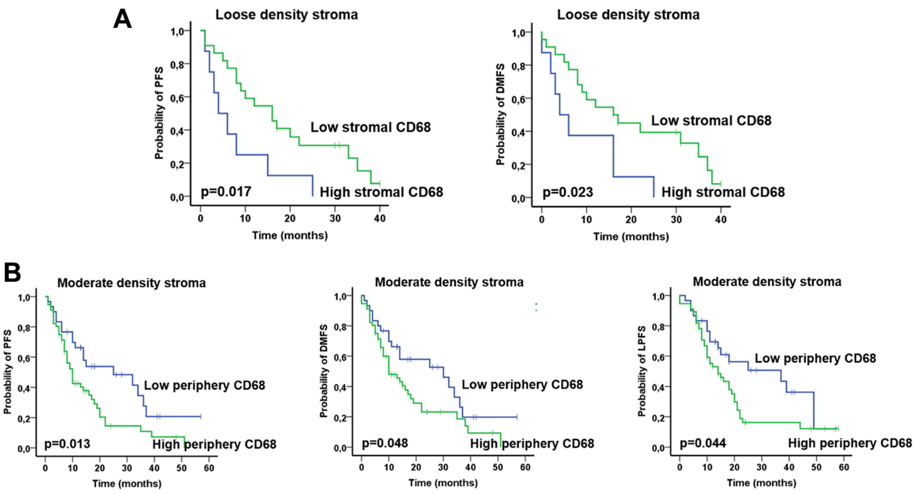

Supplementary Figure S1: Prognostic impact of (A) stromal compartment CD68 expression on progression-free survival (PFS) and distant metastases free survival (DMFS) in patients with loose stroma density, and (B) peripheral compartment CD68 expression on progression-free survival (PFS), distant metastases free survival (DMFS) and local progression-free survival (LPFS) in patients with moderate stroma density. Only significant data are shown here. Analysis was based on the dichotomized compartment CD68 score in resected patient samples (cut-off according to median value of compartment score).

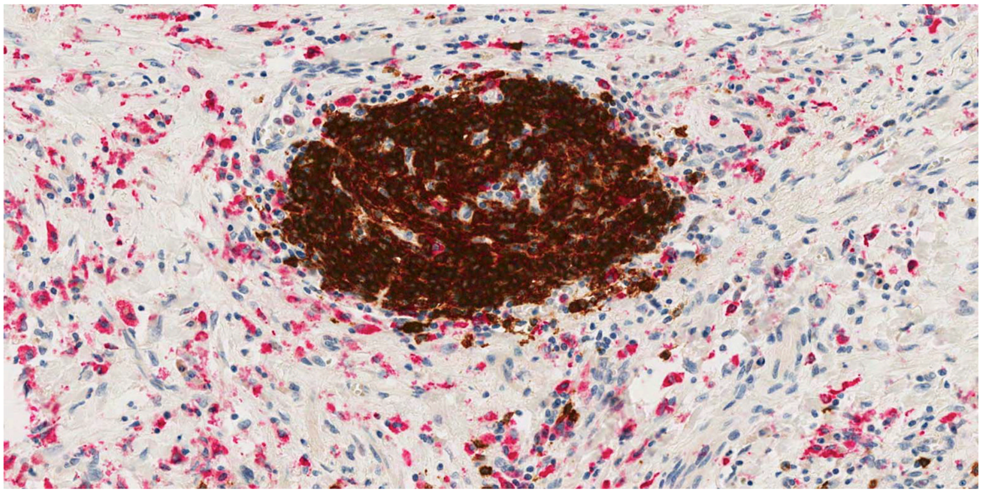

Supplementary Figure S2: Example of lymphoid aggregate following double staining for CD20+ B cells (brown) and CD68+ macrophages (purple) in a pancreatectomy section. Note that the majority of B cells are located in lymphoid aggregates with only scattered expression observed on the surrounding area. The original magnification was × 200.

Supplementary Table S1: Clinicopathological characteristics of the entire cohort (*n* = 141)

	***n* (%)**
**Age**	
< median (65 years)	62 (44%)
≥ median	79 (56%)
**Gender**	
Male	73 (51.8%)
Female	68 (48.2%)
**Tumor site**	
Head	118 (83.7%)
Other	23 (16.3%)
**pT-staging**	
pT1-2	85 (60.3%)
pT3-4	56 (39.7%)
**pN-staging**	
pN0	34 (24.1%)
pN+	107 (75.9%)
**Grading**	
G1	8 (5.7%)
G2	90 (63.8%)
G3	43 (30.5%)
**Resection margins**	
R0	53 (37.6%)
R1	88 (62.4%)
**Type of surgery**	
Whipples	91 (64.5%)
Pylorus preserving	37 (26.2%)
Total pancreatectomy	13 (9.2%)
**PNI**	
no	111 (78.7%)
yes	30 (21.3%)
**VI**	
no	51 (36.2%)
yes	90 (63.8%)
**LI**	
no	52 (36.9%)
yes	89 (63.1%)
**Chemotherapy**	
No	19 (13.5%)
1-2 cycles	30 (21.3%)
≥ 3 cycles	92 (65.2%)

Abbreviations: VI, vascular invasion; LI, lymphatic invasion; PNI, perineural/neural invasion.

Supplementary Table S2: Correlation of CA9 and CD31 with stroma density and activation

	Low CA9 *n* (%)	High CA9 *n* (%)	*p*-value	Low CD31 *n* (%)	High CD31 *n* (%)	*p*-value
**Stroma density**						
Loose	10 (14.3%)	20 (28.6%)	**0.037**	19 (27.1%)	11 (15.7%)	0.128
Moderate	43 (61.4%)	42 (60%)		42 (60.0)	43 (61.4%)	
Strong (high)	17 (24.3%)	8 (11.4%)		9 (12.9%)	16 (22.9%)	
**αSMA**						
Negative+Weak	17 (56.7%)	53 (48.2%)	0.410	14 (20.0%)	16 (22.9%)	0.680
Moderate+Strong	13 (43.3%)	57 (51.8%)		56 (80.0%)	54 (77.1%)	

Abbreviations: HR, hazard ratio; CI, confidence interval; VI, vascular invasion; LI, lymphatic invasion; PNI, perineural/neural invasion; *Significant values have been marked with bold.

Supplementary Table S3: Correlation of C68 and CD20 with stroma density and activation

	Low total CD68 *n* (%)	High total CD68 *n* (%)	*p*-value	Low total CD20 *n* (%)	High total CD20 *n* (%)	*p*-value
**Stroma density**						
Loose	18 (27.73%)	12 (15.8%)	0.175	13 (26.0%)	17 (18.7%)	0.072
Moderate	38 (58.5%)	48 (63.2%)		33 (66.0%)	53 (58.2%)	
Strong (high)	9 (13.8%)	16 (21.1%)		4 (8.0%)	21 (23.1%)	
**αSMA**						
Negative+Weak	17 (26.2%)	13 (17.1%)	0.191	6 (12.0%)	24 (26.4%)	0.054
Moderate+Strong	48 (73.8%)	63 (82.9%)		44 (88.0%)	67 (73.6%)	

Abbreviations: HR, hazard ratio; CI, confidence interval; VI, vascular invasion; LI, lymphatic invasion; PNI, perineural/neural invasion; *Significant values have been marked with bold.

Supplementary Table S4: Prognostic impact of CD68 and CD20 according to stroma activation

Marker expression (high vs low)	OS *p*-value	PFS *p*-value	LPFS *p*-value	DMFS *p*-value
Total CD68				
Absent/low SMA	0.804	0.779	0.747	0.997
Moderate/strong SMA	0.741	0.883	0.463	0.692
Total CD20				
Absent/low SMA	0.449	0.321	0.555	0.232
Moderate/strong SMA	0.380	0.739	0.673	0.857
Stromal compartment CD68				
Absent/low SMA	0.420	0.377	0.241	0.420
Moderate/strong SMA	0.738	0.789	0.695	0.592
Stromal compartment CD20				
Absent/low SMA	0.764	0.544	0.337	0.417
Moderate/strong SMA	0.432	0.362	0.411	0.971
Tumor compartment CD68				
Absent/low SMA	0.774	0.344	0.952	0.635
Moderate/strong SMA	0.262	0.236	0.122	0.111
Tumor compartment CD20				
Absent/low SMA	N/A	N/A	N/A	N/A
Moderate/strong SMA	0.563	0.224	0.447	0.792
Peripheral compartment CD68				
Absent/low SMA	0.989	0.312	0.721	0.793
Moderate/strong SMA	0.116	0.151	0.118	0.296
Peripheral compartment CD20				
Absent/low SMA	0.193	0.212	0.388	0.258
Moderate/strong SMA	0.461	0.327	0.392	0.109

Abbreviations: OS, overall survival; PFS, progression-free survival; LFFS, local failure-free survival; DMFS, distant metastases-free survival.

Supplementary Table S5: Prognostic role of CD68 and CD20 expression in the *n* = 57 patients with lymphoid aggregates

Immune marker	CD8 N (%)	FOXP3 N (%)
OS *p*-value	0.783	0.485
PFS *p*-value	0.092	0.783
LPFS *p*-value	0.835	0.835
DMFS *p*-value	0.479	0.479

Abbreviations: OS, overall survival; PFS, progression-free survival; FFS, local failure-free survival; DMFS, distant metastases-free survival.
